# A hidden Markov approach for ascertaining cSNP genotypes from RNA sequence data in the presence of allelic imbalance by exploiting linkage disequilibrium

**DOI:** 10.1186/s12859-015-0479-2

**Published:** 2015-02-22

**Authors:** Juan P Steibel, Heng Wang, Ping-Shou Zhong

**Affiliations:** 1 Department of Statistics and Probability, Michigan State University, 619 Red Cedar Road, East Lansing MI, 48824 USA; 2 Department of Animal Science, Michigan State University, East Lansing MI, 48824 USA; 3 Lyman Briggs College, Michigan State University, East Lansing MI, 48824 USA

**Keywords:** Hidden Markov model, RNA-seq data, Allelic specific expression

## Abstract

**Background:**

Allelic specific expression (ASE) increases our understanding of the genetic control of gene expression and its links to phenotypic variation. ASE testing is implemented through binomial or beta-binomial tests of sequence read counts of alternative alleles at a cSNP of interest in heterozygous individuals. This requires prior ascertainment of the cSNP genotypes for all individuals. To meet the needs, we propose hidden Markov methods to call SNPs from next generation RNA sequence data when ASE possibly exists.

**Results:**

We propose two hidden Markov models (HMMs), HMM-ASE and HMM-NASE that consider or do not consider ASE, respectively, in order to improve genotyping accuracy. Both HMMs have the advantages of calling the genotypes of several SNPs simultaneously and allow mapping error which, respectively, utilize the dependence among SNPs and correct the bias due to mapping error. In addition, HMM-ASE exploits ASE information to further improve genotype accuracy when the ASE is likely to be present.

Simulation results indicate that the HMMs proposed demonstrate a very good prediction accuracy in terms of controlling both the false discovery rate (FDR) and the false negative rate (FNR). When ASE is present, the HMM-ASE had a lower FNR than HMM-NASE, while both can control the false discovery rate (FDR) at a similar level. By exploiting linkage disequilibrium (LD), a real data application demonstrate that the proposed methods have better sensitivity and similar FDR in calling heterozygous SNPs than the VarScan method. Sensitivity and FDR are similar to that of the BCFtools and Beagle methods. The resulting genotypes show good properties for the estimation of the genetic parameters and ASE ratios.

**Conclusions:**

We introduce HMMs, which are able to exploit LD and account for the ASE and mapping errors, to simultaneously call SNPs from the next generation RNA sequence data. The method introduced can reliably call for cSNP genotypes even in the presence of ASE and under low sequencing coverage. As a byproduct, the proposed method is able to provide predictions of ASE ratios for the heterozygous genotypes, which can then be used for ASE testing.

**Electronic supplementary material:**

The online version of this article (doi:10.1186/s12859-015-0479-2) contains supplementary material, which is available to authorized users.

## Background

RNAseq is revolutionizing transcriptome analyses [1]. While RNAseq is typically used for transcript-centric analysis, where differential expression of genes or transcripts is tested between treatments or tissues [2], recently, RNAseq has been increasingly utilized for nucleotide-centric inferences such as, for coding SNP (cSNP) discovery [3], for cSNP genotyping to estimate population parameters [4] or for allelic specific expression [5,6].

ASE is particularly promising because it illuminates the genetic control of gene expression and its links to phenotypic variation [7]. In general, ASE testing is implemented through binomial or beta-binomial tests of counts of alternative alleles in reads aligned to cSNPs of interest in heterozygous individuals [8]. Some algorithms and models have been specifically tailored to perform this inference using RNAseq data [8-11], but most of them require prior ascertainment of cSNP genotypes to extract read counts for heterozygous sites or they require RNAseq or genomic sequence on parents of the individuals used for ASE testing to reliably infer cSNP genotypes. Moreover, most models do not include biological replication and assume either a single replicate or treat all biological replicates alike and collapse counts down to the nucleotide level. These assumptions may not be too restrictive in F1 crosses of inbred strains of individuals of model organisms [12] for which exhaustive sequence resources are available and biological variation is minimal, but they become more problematic for outbred populations and their crosses [13] and even for crosses of inbred lines when the purpose is to focus on individual variation in ASE for breeding [14] or population genetics inferences [15].

In the above cases, genotypes are called first from RNAseq using models designed for calling SNP from genomic sequence data [16-19], but there is a concern that extreme allelic imbalance could cause a heterozygous SNP to be mislabeled as homozygous or even not called at all, especially when coverage is low, as happens with low expressed genes [11,15]. This type of error is also present when calling SNP from pooled DNA samples where the expected allele frequency differs from 0, 0.5 and 1, as modeled in most SNP calling programs [20]. Moreover, while mislabeling a heterozygote as homozygote will not affect the estimation of the ASE ratio, it leads to loss in power. This is particularly important when working with outbred populations or their crosses, where the number of heterozygote individuals may be limited if the frequency of the minor allele is low. As a way to mitigate this problem, the use of phased haplotypes has been proposed [11], with the purpose of more reliably calling genotypes by exploiting linkage disequilibrium and minimizing the chances of missing heterozygote individuals. Exploitation of linkage disequilibrium is important because it has the potential to call SNP genotypes more accurately even with low expressed genes due to low sequence coverage.

In this paper, we concentrate on the problem of ascertaining the cSNP genotype when ASE is likely to be present. The HMM methods we propose improve genotyping accuracy by accounting for allelic imbalance, exploiting LD and allowing for mapping error. The hidden Markov approach has several advantages. First, it can model multiple SNPs simultaneously and their dependence through underlying hidden variables. Simultaneous modeling allows the HMM to make use of more data to estimate global parameters than a single SNP method. This results in increased accuracy in SNP calling especially in low expressed transcripts with low coverage. Second, the HMM is easy to implement through an Expectation-Maximization (EM) algorithm. Third, the HMM is very flexible. For example, it can be adapted to all kinds of modeling to account for individual variation in ASE ratios and sequence mapping errors. Fourth, the HMM is a likelihood based approach that can be easily used to make statistical inference. Consistency and asymptotic normality [21] can be established under some regularity conditions. The likelihood ratio approach may also be applied directly. Although HMM has been used successfully to identify copy number variations [22,23], it has not been applied to identify genotypes from RNA sequence data when allele specific expression exists. The proposed HMMs are immediately applicable after the SNPs are identified and locations are ascertained by existing software (e.g. VarScan). A comparison of existing software can be found in [18]

This paper is organized as follows. In the Methods section, we introduce HMM-ASE for calling the underlying genotype while predicting the ASE status; HMM-NASE will be introduced as a special case. In the Results section, we present simulation results for the prediction of the underlying SNP, using HMM-ASE and HMM-NASE first. Then a real data analysis is used to demonstrate the method proposed and compare it to other popular methods such as VarScan [17], BCFtools [19] and Beagle [24]. Concluding remarks are given in the Conclusion and Discussion section. We also provide Additional file 1 for additional details of the EM algorithms and some additional numerical results. A manual for the R package HMMASE can be found at http://www.stt.msu.edu/users/pszhong/HMMASE.html.

## Methods

The purpose of this Section is to introduce HMM-ASE, which can infer the underlying genotypes and predict SNP with ASE simultaneously using the RNA counts from the next generation sequence data. The introduction of HMM-NASE will be given as a special case at the end of this Section. Let **X**_*il*_=(*X*_*il*1_,*X*_*il*2_,*X*_*il*3_,*X*_*il*4_)^*T*^ be observed allele specific RNA counts of the *l*-th SNP for *l*=1,⋯,*L* and the *i*-th individual (*i*=1,⋯,*n*), where *X*_*il*1_,*X*_*il*2_,*X*_*il*3_,*X*_*il*4_ represent observed counts for *A*,*C*,*G* and *T*, respectively. Let $n_{\textit {il}}=\sum _{j=1}^{4}X_{\textit {ilj}}$ be the total counts at the *l*-th SNP of the *i*-th individual.

To simplify the notation, we will introduce the proposed HMM-ASE method for a bi-allelic SNP. The extension to other cases can be done in a similar manner. Without loss of generality, consider two possible alleles A and T. There are three possible genotypes, two homozygous AA and TT, and one heterozygous AT. For the heterozygous genotype, we also wish to predict if an allelic specific expression exists for alleles A and T. Specifically, we want to further classify the heterozygous genotypes AT into three states AT-NASE (heterozygous without ASE), AT-ASE-HIGH (heterozygous with ASE, with reads of A more than T) and AT-ASE-LOW (heterozygous with ASE, with reads of T more than A). For convenience, let *G*_*il*_ represent the (hidden) combination of genotype and allelic specific status at the position *l* (*l*=1,⋯,*L*) for the *i*-th individual where

(0.1) $$ G_{il}=\left\{ \begin{array}{cl} 1 & \text{for} ``\text{AA}^{\prime\prime};\\ 2 & \text{for} ``\mathrm{AT-NASE}^{\prime\prime};\\ 3 & \text{for} ``\mathrm{AT-ASE-HIGH}^{\prime\prime};\\ 4 & \text{for} ``\mathrm{AT-ASE-LOW}^{\prime\prime};\\ 5 & \text{for} ``\text{TT}^{\prime\prime}\\ \end{array} \right.  $$

where “AT-NASE” means the combination of genotype “AT” and non-allelic specific expression (NASE); “AT-ASE” means the combination of genotype “AT” and allelic specific expression (ASE). Given the observed RNA counts {**X**_*il*_:*i*=1,⋯,*n*;*l*=1,⋯,*L*}, we wish to predict the underlying genotypes *G*_*il*_ for all *i* and *l*. This prediction simultaneously determines the genotypes and the ASE status of each SNP.

Assume that sequence error exists in **X**_*il*_ so that all alleles are possibly observed. The read counts **X**_*il*_ are generated from a hierarchical model, which is determined by a hidden genotype *G*_*il*_ and allele specific ratio *δ*_*il*_. That is, given *n*_*il*_ and *δ*_*il*_, **X**_*il*_=(*X*_*il*1_,*X*_*il*2_,*X*_*il*3_,*X*_*il*4_)^*T*^ follows a multinomial distribution, i.e.,

(0.2) $$\begin{array}{@{}rcl@{}} \mathbf{X}_{il}|\delta_{il}& \sim & \text{Multinomial}(n_{il}, p(\delta_{il},e)); \end{array} $$

where *p*(*δ*_*il*_,*e*) represents the probability vector of multinomial distribution, *δ*_*il*_ represents the allelic specific ratio, *e* is used to account for the mapping error. Given *δ*_*il*_, the probabilities of observing a read as A, C, G or T are specified in the following probability vector

(0.3) $$ p(\delta_{il},e)=\left(\!\left(\!1-\frac{4e}{3}\right)\delta_{il}+\frac{e}{3},\frac{e}{3},\frac{e}{3},\left(\!\frac{4e}{3}-1\!\right)\delta_{il}+1-e\!\right)\!.  $$

The ASE ratios *δ*_*il*_ is a random variable that is generated from a distribution depending on *G*_*il*_=*k* in the following ways

(0.4) $$ \delta_{il}|G_{il}=k\sim\left\{ \begin{array}{ll} I_{\{\delta_{il}=1\}} & \text{for \(k=1\);}\\ I_{\{\delta_{il}=0.5\}} & \text{for \(k=2\);}\\ \mathbf{B}_{(0.5,1)}(\alpha_{1},\beta_{1}) & \text{for \(k=3\);}\\ \mathbf{B}_{(0,0.5)}(\alpha_{2},\beta_{2}) & \text{for \(k=4\);}\\ I_{\{\delta_{il}=0\}} & \text{for \(k=5\);}\\ \end{array}\right.  $$

where $I_{\{\delta _{\textit {il}}=a\}}$ represents a discrete random variable with probability mass one on point *a*, and **B**_(*S*,*U*)_(*α*,*β*) (*S*<*U*) is a rescaled beta distribution taking values within (*S*,*U*) which has a probability density function

(0.5) $$ \begin{aligned}  f_{\delta}(x)&= \frac{1}{(U-S)^{\alpha+\beta-1}\mathbf{Beta}(\alpha,\beta)}(x-S)^{\alpha-1}(U-x)^{\beta-1}\\ &\hfill\;\text{for \(S<x<U\)} \end{aligned}  $$

where **B** **e** **t** **a**(*α*,*β*) is the beta function with parameters *α* and *β*. Further, we assume that *δ*_*il*_ are independent given *G*_*il*_. As a usual HMM model, we assume the hidden states {*G*_*il*_:*l*=1,⋯,*L*} follows a Markov process to allow dependence among the observed counts **X**_*il*_s. The transition probability among underlying genotypes *G*_*il*_ is assumed to be

(0.6) $$ P\left(G_{il}=k'|G_{i(l-1)}=k\right)=a_{kk'}\;\text{for}\; k,k'=1,\cdots,M  $$

with initial probabilities *P*(*G*_*i*1_=*k*)=*π*_*ik*_ and *M*=5. One may note that the transition probabilities in (0.6) do not depend on the distances between SNPs, which motivates us to extend the transition probability matrix as a function of the distance between adjacent SNPs. The details of this extension and the associated algorithm are summarized in Additional file 1.

Denote the observed RNA counts data (incomplete) to be **X**_*i*_={**X**_*i*1_,⋯,**X**_*iL*_} for *i*=1,⋯,*n*. Then the posterior probability of *G*_*il*_=*k* given **X**={**X**_1_,⋯,**X**_*n*_}, i.e., *P*(*G*_*il*_=*k*|**X**), will be used for predicting the underlying genotypes and simultaneously infer the allelic specific status at the *l*-th SNP for the *i*-th individual. The posterior probability *P*(*G*_*il*_|**X**) can be computed by Bayes’ formula

(0.7) $$\begin{array}{*{20}l} \mathcal{L}_{i,k}(l):\!&=P(G_{il}=k|{\mathbf{X}})=\sum_{\mathbf{G}_{i}}P(G_{i}|{\mathbf{X}})I(G_{il}=k)\\ &=\sum_{\mathbf{G}_{i}}\frac{P({\mathbf{X}},G_{i})}{P({\mathbf{X}})}I(G_{il}=k) \end{array} $$

where **G**_*i*_=(*G*_*i*1_,⋯,*G*_*iL*_)^*T*^ is all the possible underlying genotypes combinations on the *L* positions.

To obtain the posterior probability in (0.7), we note the probabilities *P*(**X**,*G*_*i*_) and *P*(**X**) depend on a vector of unknown parameters ***θ***=(*α*_1_,*β*_1_,*α*_2_,*β*_2_,*e*,**A**)^*T*^, where **A**=(*a*_*kk*′_) are parameters in the transition matrix. We will use maximum likelihood estimates (MLE) to estimate ***θ***. We can find the MLEs of ***θ*** by an EM algorithm [25,26]. To this end, we introduce the following complete data corresponding to the observed data **X**,

$${\mathbf{Y}}=\{G_{il},\delta_{il}, \mathbf{X}_{il}: l=1,\cdots, L\} \;\text{for}\; i=1,\cdots,n. $$

The likelihood function for the complete data is

$$\begin{aligned}  L(\boldsymbol{\theta}|{\mathbf{Y}})&=f({\mathbf{Y}}|\boldsymbol{\theta})=f({\mathbf{X}}|\mathbf{G})f(\mathbf{G}|\boldsymbol{\theta})\\ &=\prod\limits_{i=1}^{n}\prod\limits_{l=1}^{L}f_{X}(\mathbf{X}_{il}|G_{il})\prod\limits_{i=1}^{n}\prod\limits_{l=2}^{L}a_{G_{i(l-1)},G_{il}}(\boldsymbol{\theta})\pi_{G_{i1}}(\boldsymbol{\theta}) \end{aligned} $$

where *f*_*X*_(**X**_*il*_|*G*_*il*_) is the conditional density of **X**_*il*_ given *G*_*il*_ obtained from (0.2) and (0.4) whose explicit forms can be found in the Additional file 1 and **G**=(**G**_1_,⋯,**G**_*n*_)^*T*^. It follows that the log-likelihood function of *L*(***θ***|**Y**) is given by

$$\begin{aligned} \log L(\boldsymbol{\theta}|{\mathbf{Y}})=&\;\sum\limits_{i=1}^{n}\sum\limits_{l=1}^{L}\log f_{X}(\mathbf{X}_{il}|G_{il})\\ &+\sum\limits_{i=1}^{n}\sum\limits_{l=2}^{L}\log\left\{a_{G_{i(l-1)},G_{il}}(\boldsymbol{\theta})\right\}\\ &+\sum\limits_{i=1}^{n}\log \left\{\pi_{G_{i1}}(\boldsymbol{\theta})\right\}. \end{aligned} $$

Given ***θ*** ^(*m*)^, the update ***θ*** ^(*m*+1)^ is found by maximizing *E*{log*L*(***θ***|**Y**)|**X**,***θ*** ^(*m*)^}. The details of the EM algorithm can be found in the Additional file 1. We implemented the EM algorithm by a forward and backward method [27]. Further details about the forward and backward algorithm can be found in the Additional file 1.

The HMM-NASE method could be considered as a simplification of the HMM-ASE method. The difference between HMM-NASE and HMM-ASE is that HMM-NASE does not consider the possible existence of ASE. As a result, the underlying genotypes of HMM-NASE only contain three states AA,TT and AT-NASE, which means that *G*_*il*_ in (0.1) can only have three possible values 1,2 and 5. The emission probability will be (0.3) with *k* set to be 1,2 and 5 in (0.4). Then the above forward backward algorithm is still applicable except that the unknown parameter is reduced to ***θ***=(*e*,**A**)^*T*^.

The real data analyzed in this paper were collected by [28]. The experimental procedures were approved by the All University Committee on Animal Use and Care at Michigan State University (AUF# 09/03-114-00).

## Results

### ᅟ

#### Simulation study

We performed a simulation study to demonstrate the proposed HMM-ASE and HMM-NASE methods. The underlying genotypes of SNPs were generated with the linkage disequilibrium (LD) information. Assume that LD(*d*) is the LD between two SNPs with distance *d*, which is a known function of *d*. For simplicity, in this simulation, we assume that the LD is a constant function of *d* and each SNP has only two possible alleles: either A or T. The genotypes were generated by combining two independent haplotypes.

Let *S*_*il*_ be the allele (either *A* or *T*) at the *l*-th SNP for the *i*-th individual. The marginal probabilities for *A* and *T* are *P*(*S*_*il*_=*A*)=*p*_*A*·_=*p*_·*A*_ and *P*(*S*_*il*_=*T*)=*p*_*T*·_=*p*_·*T*_ respectively. For any pair of SNPs which are next to each other in the position, the joint probability mass is defined as *P*(*S*_*il*_=*x*,*S*_*i*(*l*+1)_=*y*)=*p*_*xy*_ where *x*,*y* are either *A* or *T*.

Note that the LD(*d*) is then defined as

$$LD(d)=\frac{(p_{AA}-p_{A\cdot}p_{\cdot A})^{2}}{{(p_{A\cdot}p_{T\cdot})(p_{\cdot A}p_{\cdot T})}}. $$

Hence *p*_*AA*_,*p*_*AT*_,*p*_*TA*_ and *p*_*TT*_ can be computed once LD(*d*),*p*_*A*·_ and *p*_·*A*_ are given. We generated each side (a haplotype) of the SNP sequences independently. Both sides are generated by the following three steps:

- Set *l*=1 and generate a random variable *b*_*l*1_∼Bernoulli(*p*_*A*·_). If *b*_*il*_=1 then we set *S*_*il*_=*A*, otherwise *S*_*il*_=*T*.
- Let *l*=*l*+1. Generating allele at *l*+1 position conditional on the *l* position. Namely, *P*(*S*_*i*(*l*+1)_=*A*|*S*_*il*_=*x*)=*p*_*xA*_/*p*_*x*·_, where *x* could be either *A* or *T*.
- Repeating step (b) until we get *L* SNPs.

We then generated total read counts $n_{\textit {il}}=\sum _{k=1}^{4} X_{\textit {ilk}}$ from a Negative Binomial (NB) distribution independently for at each SNP *l*=1,⋯,*L* and individual *i*=1,⋯,*n*. Conditional on the underlying genotype and the total RNA counts, we generated the allele specific RNA counts **X**_*il*_ through the hierarchical model given in (0.2) through (0.4). For illustration purpose, we considered a data set that was generated only by 4 underlying states in the simulation. Namely, AA, AT-NASE, AT-ASE-HIGH and TT, where the distribution of the allele specific ratio *δ*_*il*_ for the AT-ASE-HIGH state was changed to a Beta(*α*,*β*) distribution where we set *α*=30,*β*=10 such that the mode and center of the beta distribution is concentrated around 0.75.

The following scenarios were used in the simulation. We designed three different numbers of individuals (*n*): 6, 12 and 24, and two different numbers of SNP (*L*): 10 and 100. For each of the above six individual/SNP combinations, the total number of reads were simulated from negative binomial distributions NB(*λ*,0.4) with five different *λ* values: 8, 16, 24, 32 and 56 as well as two different values for LD: 0.5 and 0.8. We did 10 replications for each of the above combinations. We measured the performance of the proposed method by empirical false discovery rate (EFDR) and empirical false negative rate (EFNR), where were defined, respectively, as

$$\begin{aligned} \text{EFDR}&=\frac{\text{\# Homozygotes called heterozygotes}}{\text{Total \# of called heterozygous}};\\ \text{EFNR}&=\frac{\text{\# Heterozygotes called homozygotes}}{\text{Total \# of called homozygous}}. \end{aligned} $$

**Figure 1:**
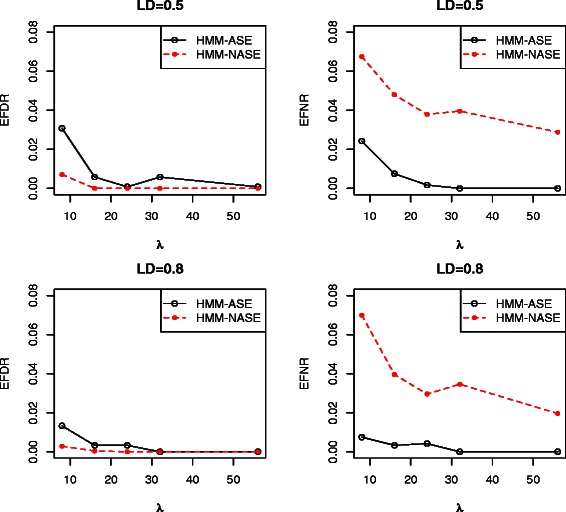
**A comparison of the EFDR and EFNR for the HMM-ASE vs HMM-NASE for** ***n=24,L=10*** **with LD=** ***0.5*** **and 0.8.**

The proposed HMM-ASE and HMM-NASE were applied to the above scenarios. Table 1 and Table 2 summarize the EFDR and EFNR for both methods in the case with 10 SNPs. The first and second columns of both tables represent the values of *λ* and LD. The larger the value of *λ*, the larger the average of the RNA counts. On one hand, HMM-ASE and HMM-NASE share some similarity. By increasing the value of *λ*, the EFDR and EFNR of both HMMs were smaller with lower variability (smaller standard deviation). Increasing the LD value led to better predictions, which shows that both HMMs made use of the LD information in predicting genotypes. Specifically, one can see that the EFDR and EFNR rates were improved with increased LD when *λ* is relative small. On the other hand, the EFNR rates of HMM-NASE in Table 2 were almost always consistently larger than the EFNR rates from HMM-ASE in the same table while the EFDR (Table 1) of HMM-ASE were slightly higher than those of HMM-NASE when *λ* is relative small. This demonstrates that HMM-ASE has better sensitivity than HMM-NASE in heterozygote SNP genotypes from RNAseq data when ASE is likely present because ASE is considered in HMM-ASE. To better illustrate these two methods, Figure 1 compared the EFDR and EFNR for HMM-ASE and HMM-NASE methods when *n*=24.

**Table 1:** **EFDR (homozygotes called heterozygotes) in blocks of 10 SNP using HMM-ASE and HMM-NASE**

**(** ***λ*** **, LD)**		**Number of individuals**		
		**6**	**12**	**24**
(8,0.5)	ASE	0.0167 (0.017)	0.0117 (0.010)	0.0308 (0.020)
	NASE	0.0033 (0.007)	0.0050 (0.006)	0.0071 (0.004)
(8,0.8)	ASE	0.0000 (0.000)	0.0150 (0.007)	0.0133 (0.005)
	NASE	0.0017 (0.005)	0.0050 (0.006)	0.0029 (0.004)
(16,0.5)	ASE	0.0100 (0.009)	0.0000 (0.000)	0.0058 (0.004)
	NASE	0.0017 (0.005)	0.0008 (0.003)	0.0000 (0.000)
(16,0.8)	ASE	0.0033 (0.008)	0.0017 (0.004)	0.0033 (0.004)
	NASE	0.0000 (0.000)	0.0008 (0.003)	0.0004 (0.001)
(24,0.5)	ASE	0.0000 (0.000)	0.0033 (0.005)	0.0008 (0.002)
	NASE	0.0000 (0.000)	0.0000 (0.000)	0.0000 (0.000)
(24,0.8)	ASE	0.0000 (0.000)	0.0000 (0.000)	0.0033 (0.004)
	NASE	0.0000 (0.000)	0.0000 (0.000)	0.0000 (0.000)
(32,0.5)	ASE	0.0000 (0.000)	0.0000 (0.000)	0.0058 (0.006)
	NASE	0.0000 (0.000)	0.0000 (0.000)	0.0000 (0.000)
(32,0.8)	ASE	0.0000 (0.000)	0.0017 (0.004)	0.0000 (0.000)
	NASE	0.0000 (0.000)	0.0000 (0.000)	0.0000 (0.000)
(56,0.5)	ASE	0.0000 (0.000)	0.0000 (0.000)	0.0008 (0.002)
	NASE	0.0000 (0.000)	0.0000 (0.000)	0.0000 (0.000)
(56,0.8)	ASE	0.0000 (0.000)	0.0000 (0.000)	0.0000 (0.000)
	NASE	0.0000 (0.000)	0.0000 (0.000)	0.0000 (0.000)

Standard deviations are included in parenthesis.

**Table 2:** **EFNR (heterozygotes called homozygotes) in blocks of 10 SNP using HMM-ASE and HMM-NASE**

**(** ***λ*** **, LD)**		**Number of individuals**		
		**6**	**12**	**24**
(8,0.5)	ASE	0.0367 (0.018)	0.0217 (0.013)	0.0242 (0.028)
	NASE	0.0833 (0.038)	0.0733 (0.019)	0.0675 (0.015)
(8,0.8)	ASE	0.0100 (0.009)	0.0033 (0.005)	0.0075 (0.005)
	NASE	0.0450 (0.046)	0.0533 (0.026)	0.0700 (0.020)
(16,0.5)	ASE	0.0133 (0.014)	0.0067 (0.004)	0.0075 (0.004)
	NASE	0.0567 (0.036)	0.0433 (0.025)	0.0479 (0.016)
(16,0.8)	ASE	0.0067 (0.015)	0.0067 (0.011)	0.0033 (0.004)
	NASE	0.0467 (0.046)	0.0367 (0.023)	0.0396 (0.011)
(24,0.5)	ASE	0.0033 (0.008)	0.0000 (0.000)	0.0017 (0.002)
	NASE	0.0233 (0.025)	0.0308 (0.020)	0.0379 (0.014)
(24,0.8)	ASE	0.0033 (0.008)	0.0000 (0.000)	0.0042 (0.007)
	NASE	0.0383 (0.022)	0.0392 (0.026)	0.0296 (0.016)
(32,0.5)	ASE	0.0000 (0.000)	0.0033 (0.005)	0.0000 (0.000)
	NASE	0.0300 (0.019)	0.0375 (0.021)	0.0396 (0.019)
(32,0.8)	ASE	0.0000 (0.000)	0.0000 (0.000)	0.0000 (0.000)
	NASE	0.0417 (0.043)	0.0208 (0.013)	0.0346 (0.018)
(56,0.5)	ASE	0.0033 (0.008)	0.0000 (0.000)	0.0000 (0.000)
	NASE	0.0233 (0.014)	0.0275 (0.013)	0.0288 (0.009)
(56,0.8)	ASE	0.0033 (0.008)	0.0000 (0.000)	0.0000 (0.000)
	NASE	0.0200 (0.019)	0.0200 (0.015)	0.0196 (0.009)

Standard deviations are included in parentheses.

Finally, we assessed the effect of increasing the size of the SNP block on HMM-ASE and HMM-NASE results. We repeated the simulations using 100 SNP blocks. The results are presented in Tables 3 and 4. For the LD structure, coverage and sample sizes used in this simulation, there was virtually no significant difference in error rates compared to the results using a smaller SNP block. This suggests that small SNP blocks are as efficient as larger blocks in utilizing the advantage of LD. Thus, we prefer using small blocks to reduce the computation load. In addition, we also observed that the number of individuals used in the simulation only had minor effects on the EFDR and EFNR with differences well within the range of standard errors.

**Table 3:** **The EFDR (homozygous called heterozygous) and EFNR (heterozygous called homozygous) with 100 SNPs for HMM-ASE**

**(** ***λ*** **, LD)**	**Number of individuals**		
	**6**	**12**	**24**
		**EFDR**	
(8,0.5)	0.0163 (0.009)	0.0163 (0.003)	0.0230 (0.004)
(8,0.8)	0.0067 (0.003)	0.0050 (0.002)	0.0068 (0.003)
(16,0.5)	0.0050 (0.002)	0.0053 (0.001)	0.0068 (0.001)
(16,0.8)	0.0033 (0.002)	0.0025 (0.001)	0.0030 (0.001)
(24,0.5)	0.0027 (0.002)	0.0017 (0.001)	0.0030 (0.002)
(24,0.8)	0.0017 (0.002)	0.0002 (0.000)	0.0009 (0.001)
(32,0.5)	0.0017 (0.002)	0.0008 (0.001)	0.0008 (0.001)
(32,0.8)	0.0007 (0.001)	0.0008 (0.001)	0.0008 (0.000)
(56,0.5)	0.0007 (0.001)	0.0000 (0.000)	0.0008 (0.000)
(56,0.8)	0.0003 (0.001)	0.0002 (0.000)	0.0002 (0.000)
		**EFNR**	
(8,0.5)	0.0230 (0.003)	0.0218 (0.007)	0.0138 (0.003)
(8,0.8)	0.0113 (0.005)	0.0088 (0.004)	0.0071 (0.003)
(16,0.5)	0.0080 (0.004)	0.0063 (0.003)	0.0047 (0.002)
(16,0.8)	0.0033 (0.001)	0.0032 (0.002)	0.0028 (0.001)
(24,0.5)	0.0017 (0.002)	0.0028 (0.002)	0.0022 (0.001)
(24,0.8)	0.0020 (0.001)	0.0017 (0.001)	0.0010 (0.001)
(32,0.5)	0.0013 (0.001)	0.0005 (0.001)	0.0011 (0.001)
(32,0.8)	0.0003 (0.001)	0.0005 (0.001)	0.0004 (0.000)
(56,0.5)	0.0003 (0.001)	0.0005 (0.001)	0.0001 (0.000)
(56,0.8)	0.0003 (0.001)	0.0002 (0.000)	0.0000 (0.000)

Their standard deviations are shown in the parentheses.

**Table 4:** **The EFDR (homozygous called heterozygous) and EFNR (heterozygous called homozygous) with 100 SNPs for HMM-NASE**

**(** ***λ*** **, LD)**	**Number of individuals**		
	**6**	**12**	**24**
		**EFDR**	
(8,0.5)	0.0053 (0.005)	0.0035 (0.001)	0.0045 (0.001)
(8,0.8)	0.0025 (0.002)	0.0015 (0.001)	0.0020 (0.001)
(16,0.5)	0.0005 (0.001)	0.0003 (0.001)	0.0005 (0.000)
(16,0.8)	0.0002 (0.001)	0.0002 (0.000)	0.0001 (0.000)
(24,0.5)	0.0000 (0.000)	0.0000 (0.000)	0.0000 (0.000)
(24,0.8)	0.0000 (0.000)	0.0000 (0.000)	0.0000 (0.000)
(32,0.5)	0.0000 (0.000)	0.0000 (0.000)	0.0000 (0.000)
(32,0.8)	0.0000 (0.000)	0.0000 (0.000)	0.0000 (0.000)
(56,0.5)	0.0000 (0.000)	0.0000 (0.000)	0.0000 (0.000)
(56,0.8)	0.0000 (0.000)	0.0000 (0.000)	0.0000 (0.000)
		**EFNR**	
(8,0.5)	0.0728 (0.015)	0.0740 (0.009)	0.0701 (0.005)
(8,0.8)	0.0533 (0.011)	0.0498 (0.014)	0.0520 (0.005)
(16,0.5)	0.0557 (0.012)	0.0466 (0.009)	0.0467 (0.005)
(16,0.8)	0.0358 (0.010)	0.0364 (0.008)	0.0401 (0.009)
(24,0.5)	0.0412 (0.011)	0.0398 (0.007)	0.0407 (0.003)
(24,0.8)	0.0347 (0.006)	0.0298 (0.008)	0.0305 (0.004)
(32,0.5)	0.0280 (0.007)	0.0338 (0.004)	0.0313 (0.005)
(32,0.8)	0.0292 (0.010)	0.0304 (0.007)	0.0285 (0.003)
(56,0.5)	0.0257 (0.007)	0.0268 (0.004)	0.0265 (0.003)
(56,0.8)	0.0203 (0.008)	0.0218 (0.003)	0.0213 (0.002)

Their standard deviations shown in the parenthesis.

#### Simulation conditional on haplotypes from real data

To make the haplotype structures in the simulation data more realistic, we randomly selected hayplotype structures from a real data in the pig resource population [28] to create the genotypes. Conditional on the genotypes, we generated the counts data using the same methods as those in Tables 1, 2, 3 and 4. Results of the EFDR and EFNR of the HMM-ASE and HMM-NASE methods are shown in Table 5. HMM-ASE still maintained a low level EFDR and EFNR indicating that the HMM-ASE method is robust to the change of underlying haplotype structures. But the EFNR of the HMM-NASE method was higher in Table 5, because HMM-NASE did not account for the ASE, which exists in the simulated data. To confirm this, we further generated counts without ASE (ASE ratios =0.5), the results are summarized in Table 6. Both the HMM-ASE and HMM-NASE methods performed well in this case, suggesting that the HMM-NASE method is robust to the change of haplotype structures but not to the existence of ASE. This confirms the importance of developing the HMM-ASE method.

**Table 5:** **The EFDR (homozygous called heterozygous) and EFNR (heterozygous called homozygous) with 100 SNPs using the haplotype structures from real data**

***λ***		**Number of individuals**		
		**6**	**12**	**24**
		EFDR		
8	ASE	0.0784 (0.0234)	0.0729 (0.0093)	0.0752 (0.0089)
	NASE	0.0235 (0.0150)	0.0276 (0.0119)	0.0267 (0.0111)
16	ASE	0.0328 (0.0099)	0.0338 (0.0088)	0.0331 (0.0086)
	NASE	0.0016 (0.0027)	0.0028 (0.0036)	0.0019 (0.0017)
24	ASE	0.0153 (0.0070)	0.0137 (0.0064)	0.0164 (0.0054)
	NASE	0.0012 (0.0026)	0.0008 (0.0013)	0.0004 (0.0006)
32	ASE	0.0118 (0.0087)	0.0110 (0.0031)	0.0076 (0.0033)
	NASE	0.0000 (0.0000)	0.0000 (0.0000)	0.0001 (0.0003)
56	ASE	0.0022 (0.0033)	0.0030 (0.0028)	0.0019 (0.0011)
	NASE	0.0000 (0.0000)	0.0000 (0.0000)	0.0000 (0.0000)
		**EFNR**		
8	ASE	0.0418 (0.0169)	0.0479 (0.0129)	0.0502 (0.0098)
	NASE	0.2161 (0.0533)	0.2084 (0.0434)	0.2075 (0.0410)
16	ASE	0.0239 (0.0095)	0.0214 (0.0109)	0.0211 (0.0056)
	NASE	0.1888 (0.0517)	0.1986 (0.0455)	0.1762 (0.0360)
24	ASE	0.0161 (0.0077)	0.0164 (0.0093)	0.0143 (0.0032)
	NASE	0.1523 (0.0584)	0.1788 (0.0503)	0.1577 (0.0246)
32	ASE	0.0060 (0.0071)	0.0079 (0.0063)	0.0088 (0.0036)
	NASE	0.1434 (0.0839)	0.1367 (0.0507)	0.1368 (0.0227)
56	ASE	0.0040 (0.0057)	0.0031 (0.0028)	0.0025 (0.0012)
	NASE	0.1339 (0.1005)	0.1178 (0.0348)	0.1332 (0.0287)

Their standard deviations shown in the parenthesis.

**Table 6:** **The EFDR (homozygous called heterozygous) and EFNR (heterozygous called homozygous) with 100 SNPs using the haplotype structures from real data**

***λ***		**Number of individuals**		
		**6**	**12**	**24**
		**EFDR**		
8	ASE	0.0222 (0.0100)	0.0264 (0.0091)	0.0243 (0.0038)
	NASE	0.0250 (0.0064)	0.0313 (0.0103)	0.0297 (0.0060)
16	ASE	0.0026 (0.0032)	0.0023 (0.0020)	0.0043 (0.0033)
	NASE	0.0023 (0.0025)	0.0023 (0.0027)	0.0040 (0.0033)
24	ASE	0.0006 (0.0013)	0.0003 (0.0008)	0.0006 (0.0005)
	NASE	0.0006 (0.0013)	0.0000 (0.0000)	0.0003 (0.0005)
32	ASE	0.0000 (0.0000)	0.0002 (0.0006)	0.0004 (0.0005)
	NASE	0.0000 (0.0000)	0.0000 (0.0000)	0.0000 (0.0000)
56	ASE	0.0000 (0.0000)	0.0000 (0.0000)	0.0000 (0.0000)
	NASE	0.0000 (0.0000)	0.0000 (0.0000)	0.0000 (0.0000)
		**EFNR**		
8	ASE	0.0375 (0.0224)	0.0295 (0.0090)	0.0259 (0.0072)
	NASE	0.0322 (0.0193)	0.0251 (0.0080)	0.0219 (0.0048)
16	ASE	0.0028 (0.0032)	0.0030 (0.0024)	0.0033 (0.0020)
	NASE	0.0037 (0.0043)	0.0027 (0.0021)	0.0028 (0.0019)
24	ASE	0.0002 (0.0008)	0.0000 (0.0000)	0.0006 (0.0006)
	NASE	0.0000 (0.0000)	0.0000 (0.0000)	0.0006 (0.0007)
32	ASE	0.0000 (0.0000)	0.0000 (0.0000)	0.0000 (0.0000)
	NASE	0.0003 (0.0011)	0.0002 (0.0005)	0.0000 (0.0000)
56	ASE	0.0000 (0.0000)	0.0000 (0.0000)	0.0000 (0.0000)
	NASE	0.0000 (0.0000)	0.0000 (0.0000)	0.0000 (0.0000)

The data were generated without ASE, namely, all the heterozygous genes have ASE ratio 0.5. Their standard deviations shown in the parenthesis.

#### Real data analysis

**Assessing accuracy of heterozygote calling rates** Called cSNP genotypes were compared to gold-standards or true genotypes. In the simulation, the true genotype was readily available. For the real data analysis, genotypes obtained from a DNA SNP chip were used as a gold standard to evaluate the performance of cSNP calling and genotyping. The EFDR and Sensitivity were computed to assess the accuracy of genotype calling where

$$\text{Sensitivity} =\frac{\text{\# Heterozygotes called heterozygotes}}{\text{Total \# of heterozygotes}}. $$

These measures are especially relevant when the intent of genotype calling is to perform ASE testing, because they focus on key heterozygous genotypes. EFDR and Sensitivity can be computed globally across all sites and individuals on a cSNP site basis.

**Comparison with alternative methods** We applied the proposed HMMs, HMM-ASE and HMM-NASE, to a real RNAseq dataset and compared SNP genotype calls with those from VarScan, SAMtools+BCFtools and BEAGLE, well-known methods for SNP and mutation calling from sequence data. RNAseq data were available for 24 female pigs from an F2 cross of Duroc and Pietrain in our pig resource population [29-32]. Pig breeds are outbred and show substantial variation in allele frequency, high linkage disequilibrium within breed and limited phase agreement between breeds [33]. These animals were part of a larger transcriptional profiling study [34] and had been selected because they showed extreme phenotypes for loin eye area (a trait of economic value) compared to their litter mates. SNP chip data were available from the 60K illumina chip [35] from a recent study. Genotype data from the chip were treated as a gold standard against which cSNP called with RNAseq were validated. RNA from each sample was reverse transcribed into cDNA, fragmented and labeled to generate 24 barcoded libraries that were sequenced on an Illumina HiSeq 2000 (100 bp, paired-end reads). Each library was sequenced in four lanes with the raw read data consisting of 96 pairs of fastq files (4 per sample) containing approximately 15million short-reads (100 bp) each. Those fastq files were pre-processed using FASTX toolkit (http://hannonlab.cshl.edu/fastx_toolkit/) and FASTQC (http://www.bioinformatics.babraham.ac.uk/projects/fastqc/) to assess read quality. Then, Tophat [36] was used for mapping the reads to the reference genome (Sus scrofa 10.2.69 retrieved from the Ensembl database) using an index generated by Bowtie2 [37]. The aligned records were stored in BAM/SAM format [38]. Alignment statistics and base coverage were calculated for each file using SAMTools [38]. Initially coding SNP discovery and genotyping were done with VarScan [17]. First, a base alignment file (.mpileup) for each covered position was obtained for each chromosome using the mpileup option of SAMTools [19] and subsequently VarScan [17] was used to call genotypes and count reads mapping to each segregating allele. We focused on chromosome 13 and extracted counts of reads agreeing with reference (R) or alternative (A) allele with respect to the reference genome at putative 5364 cSNP discovered by VarScan, which included 65 SNPs represented in the 60K chip for which we had reliable genotype data. We segmented the SNP data into 65 brackets that included each of those SNP and their surrounding cSNPs. Each bracket was analyzed separately with our program because we expect that only closely linked cSNPs will benefit from our multi-SNP HMM model.

There were a total of 1560 genotypes to impute (24 animals and 65 SNPs), 591 heterozygotes and 969 homozygotes. VarScan (Table 7) did not impute any homozygotes as heterozygotes (EFDR = 0), but it only correctly identified 449 of the 591 heterozygotes (Sensitivity = 0.76). This drop in sensitivity to detect heterozygotes was accounted for by the non-call rate (64/591 =0.11) and wrongly calling 78 heterozygotes as homozygotes (0.14). The HMM-ASE and HMM-NASE had an EFDR =0.015 (9/(571 + 9)) and 2/(570 + 2) =0.0035, respectively. But the sensitivities were 1.0 (detected all Heterozygotes) and 0.998, respectively if the restriction of having at least one read was imposed. Remarkably, even some genotypes without any reads were imputed correctly due to exploitation of zygotic disequilibrium. In this particular data set, HMM-ASE did not show additional advantage by considering ASE effects, indicating that modeling dependence is more important than modeling ASE. We also called cSNPs using two widely used algorithms: SAMtools+BCFtools and SAMtools+BCFtools+Beagle. SAMtools+BCFtools is probably the most commonly used algorithm for calling SNP, it calls SNP genotypes independently and its likelihood function assumes no ASE. When read counts are very low, SAMtools+BCFtools may not call SNP genotypes. Alternatively, BEAGLE uses the output from the previous algorithm and performs SNP calling by accounting for LD. Table 7 shows that these two methods were very similar to the proposed methods in terms of EFDR, which were 0.007 and 0.010, respectively. These two methods performed slightly better than HMM-ASE and HMM-NASE for SNPs with zero counts. But the proposed methods were slightly better than the SAMtools+BCFtools and Beagle methods for SNPs with non-zero counts (See Table S.2 in Section 3 of the Additional file 1). Since the performance of HMM-ASE, HMM-NASE, SAMtools+BCFtools and Beagle methods were very similar in discovering heterozygous SNPs, we decided to proceed with HMM-ASE analysis after assigning non-calls to those genotypes without any read.

**Table 7:** **Contingency tables of genotype calling with five methods (columns) versus actual genotypes (rows)**

**Actual**	**VarScan Genotype**			**HMM-ASE**			**HMM-NASE**		
genotype	He	Ho	NC	He	Ho	NC	He	Ho	NC
						(Reads =0)			(Reads =0)
He	449	78	64	**571**	0	20	**570**	1	20
Ho	0	886	83	9	**914**	46	2	**921**	46
**Actual**	**SAMTOOLS + BCFTOOLS**			**BEAGLE**					
genotype	He	Ho	NC	He	Ho	NC			
He	**576**	9	6	**583**	8	0			
Ho	4	**957**	8	6	**963**	0			

Values in bold represent counts of correct calls. The other values are incorrect calls or Non-called (NC).

**Figure 2:**
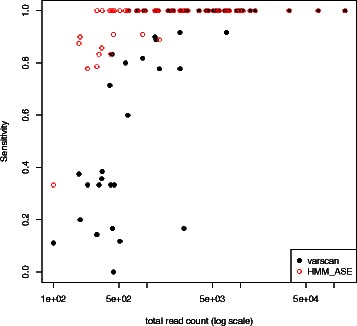
**Sensitivity (proportion of Heterozygotes genotypes called by each program) as a function of total read count.**

**Figure 3:**
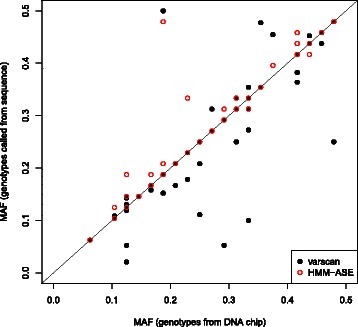
**Estimated minor allele frequency using genotypes from DNA chip or from called cSNP.**

Upon a SNP-by-SNP analysis of genotype calls, the low sensitivities obtained with the two programs (HMM-ASE and VarScan) for some markers can be largely explained by low coverage (Figure 2). In our HMM-ASE model, having at least 200 total reads (across all 24 individuals) produced sensitivities over 0.8 but the effect of these errors in final inferences is not clear. For example, if inferred genotypes are used for ASE, the effect of missing a heterozygote is lower power, while the effect of incorrectly imputing a homozygote as heterozygote could be biases in the estimated ASE (and false positive rate of ASE tests). Conversely, the two errors could potentially cancel out when using genotypes to estimate minor allelic frequency (MAF). Consequently, we proceeded to estimate MAF using ascertained genotypes and confirmed that HMM-ASE estimated MAF very precisely (Figure 3) with a correlation of 0.94 with DNA chip based estimates.

**Figure 4:**
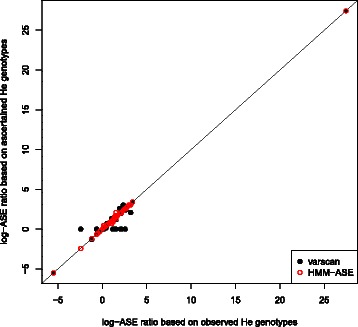
**log-ASE ratio of fitting beta-binomial model on counts from Heterozygous individuals.** Heterozygous status was either taken from chip data (x-axis) or from genotypes called from sequence data (y-axis).

**Figure 5:**
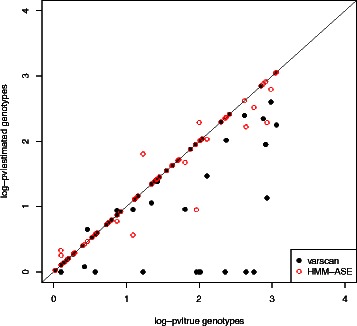
***log10*** **(p-value) of fitting beta-binomial model on counts from Heterozygous individuals.** Heterozygous status was either taken from chip data (x-axis) or from genotypes called from sequence data (y-axis).

In order to assess the effect of genotype ascertainment on estimation and testing of ASE, we used heterozygous genotypes (either observed with chip or ascertained) and fit a beta-binomial model [39] to read counts. In fact, when working with HMM-ASE this would not be necessary because our method produced estimates of ASE parameters, but we used the beta-binomial model in order to separately assess the effect of genotype calling errors. In Figure 4 we observe that using genotypes ascertained with the HMM-ASE produced very accurate estimates of ASE. On the other hand, using heterozygote genotypes ascertained with VarScan also produced good agreement with those from chip data, except when the sensitivity was very low either because of calling heterozygotes as homozygotes or because of non-calling a genotype (horizontal points close to zero). These effects were even more obvious when looking at associated p-values. In that case, missing heterozygotes from the single SNP analysis program (in this case VarScan) substantially reduced significance (Figure 5).

## Conclusion and discussion

In this paper, we present HMM methods to call SNP genotypes in the presence of allelic imbalance by exploiting zygotic disequilibrium. In its present form, HMM-ASE and HMM-NASE require that cSNP locations have been previously ascertained and conditional on those it can accurately call their genotypes. Our program is particularly useful for cSNP genotyping after a SNP discovery step has been applied [18]. This is important because while many programs are tailored for cSNP discovery [18] and genotyping assuming allelic balance in heterozygotes, there is a need for accurate genotype calling by exploiting linkage disequilibrium in the presence of ASE in individuals from outbred populations [15]. HMM-ASE and HMM-NASE can use read counts of 4 bases or pre-filtered biallelic counts. The biallelic option was used in the real data case because previous cSNP discovery using VarScan produced counts of reference and alternative alleles. A similar pre-processing step can be performed with a number of available programs [18]. The strength of HMM compared to other methods is that it can reliably call heterozygous cSNP genotypes even in the presence of ASE and under low sequencing coverage. Furthermore, by comparing to DNA-chip genotypes, genotypes produced using the proposed methods resulted in good estimates of ASE and MAF. This is important in population genetics that can use low-coverage sequence of many individuals in order to accurately estimate MAF, linkage disequilibrium and other population genomics parameters [33]. Another potential use of genotypes obtained from HMMs is to perform ASE testing [13]. Although not extensively studied in this first paper, HMM-ASE could be used to derive not only the cSNP genotypes but their ASE ratios. In its current form, HMM-ASE integrates out such information when calling genotypes, but further work in this area is warranted.

An important part of implementing our HMM algorithm in HMM-ASE and HMM-NASE consists of segmenting the SNPs in groups that are tractable and informative. From the simulation study and real data analysis, we found that the HMM-NASE was very robust in terms of group segmentation but HMM-ASE was slightly more sensitive to the number of SNPs and the length of the segment. In particular, EFDR (genotyping homozygotes as heterozygotes) was slightly lower when the segment was 1kb long compared to 4 kb long. We experimented with many criteria to partition the SNPs in the real data set and found that the inferences were robust to the number of SNPs in the segment for a range of 2 to 25 SNPs over a 1/2 kb to 2 kb long segments. We only observed one SNP within a 1 kb segment containing 35 SNPs had slightly higher EFDR. Further inspection of the segment indicated that this region likely included SNPs from several transcripts and that the zygotic disequilibrium seemed to be low for SNPs on different transcripts. A possibility to mitigate this problem could be to group SNP by transcript by using bioinformatics tools such as the ensemble variation API [40]. Since this problem was sporadic (one segment) in our data set we believe that such an approach was not needed.

In summary, in this paper we present and evaluate an algorithm for calling SNP genotypes in the presence of allelic imbalance by exploiting linkage disequilibrium. The method is particularly suitable for calling cSNP from low-coverage RNA-seq data and the resulting genotypes show good properties for estimation of genetic parameters and allelic ratios. We provide HMMASE, an R package to implement the proposed algorithm (http://www.stt.msu.edu/users/pszhong/HMMASE.html). Our algorithm performed better than VarScan and similarly to BCFtools and Beagle, indicating that the joint modeling of ASE and LD recovered important information although our algorithm did not use haplotypes information. Furthermore, our promising results encourage further research on extending the algorithm to incorporate haplotype structures and performing the ASE testing.

## Availability of supporting data

The data set and the R package HMMASE supporting the results of this article are available in http://www.stt.msu.edu/users/pszhong/HMMASE.html.

## Additional file

Additional file 1
**Supplemental.** Contains a detailed algorithm of the proposed HMM-ASE algorithm **(Section 1)**, with an extension to the case with distance dependent transition matrix **(Section 2)**. In **Section 3**, we include some additional real data analysis results using distance dependent transition matrix and the comparison with BCFtools and Beagle after excluding the zero counts in the data set. In **Section 4**, two additional tables for simulation studies with haplotypes generated from the real data are also included.

## References

[1] Wang Z, Gerstein M, Snyder M (2009). RNA-Seq: a revolutionary tool for transcriptomics. Nat Rev Genet.

[2] Robinson MD, McCarthy DJ, Smyth GK (2010). edgeR: a Bioconductor package for differential expression analysis of digital gene expression data. Bioinformatics.

[3] Cánovas A, Rincon G, Islas-Trejo A, Wickramasinghe S, Medrano JF (2010). SNP discovery in the bovine milk transcriptome using RNA-Seq technology. Mammalian Genome.

[4] Nielsen R, Korneliussen T, Albrechtsen A, Li Y, Wang J (2012). SNP calling, genotype calling, and sample allele frequency estimation from New-Generation sequencing data. PLoS One.

[5] Montgomery S, Sammeth M, Gutierrez-Arcelus M, Lach R, Ingle C, Nisbett J (2010). Transcriptome genetics using second generation sequencing in a Caucasian population. Nature.

[6] Pickrell J, Marioni Pai A, Degner J, Engelhardt B, Nkadori E, Veyrieras J (2010). Understanding mechanisms underlying human gene expression variation with RNA sequencing. Nature.

[7] Pastinen T (2010). Genome-wide allele-specific analysis: insights into regulatory variation. Nat Rev Genet.

[8] Sun W (2012). A statistical framework for eQTL mapping using RNA-seq data. Biometrics.

[9] Pandey R, Franssen S, Futschik A, Schlotterer C (2013). Allelic imbalance metre (Allim), a new tool for measuring allele-specific gene expression with RNA-seq data. Mol Ecol Resour.

[10] Rozowsky J, Abyzoy A, Wang J, Alves P, Raha D, Harmanci A (2011). AlleleSeq: analysis of allele-specific expression and binding in a network framework. Mol Syst Biol.

[11] Turro E, Su S, Gonçalves A, Coin L, Richardson S, Lewin A (2011). Haplotype and isoform specific expression estimation using multi-mapping RNA-seq reads. Genome Biol.

[12] Skelly D, Johansson M, Madeoy J, Wakefield J, Akey J (2011). A powerful and flexible statistical framework for testing hypotheses of allele-specific gene expression from RNA-seq data. Genome Res.

[13] Ernst CW, Steibel JP (2013). Molecular advances in QTL discovery and application in pig breeding. Trends Genet.

[14] Perumbakkam Muir W, Black-Pyrkosz A, Okimoto R, Cheng H (2013). Comparison and contrast of genes and biological pathways responding to Marek’s disease virus infection using allele-specific expression and differential expression in broiler and layer chickens. BMC Genomics.

[15] Singhal S (2013). De novo transcriptomic analyses for non-model organisms: an evaluation of methods across a multi-species data set. Mol Ecol Resources.

[16] DePristo M, Banks E, Poplin R, Garimella K, Maguire J, Hartl C (2011). A framework for variation discovery and genotyping using next-generation DNA sequencing data. Nat Genet.

[17] Koboldt D, Chen K, Wylie T, Larson D, McLellan M, Mardis E (2009). VarScan: variant detection in massively parallel sequencing of individual and pooled samples. Bioinformatics.

[18] You N, Murillo G, Su X, Zeng X, Ning K, Zhang S (2012). SNP calling using genotype model selection on high-throughput sequencing data. Bioinformatics.

[19] Li H (2011). A statistical framework for SNP calling, mutation discovery, association mapping and population genetical parameter estimation from sequencing data. Bioinformatics.

[20] Raineri E, Ferretti L, Esteve-Codina A, Nevado B, Heath S, Pérez-Enciso M (2012). SNP calling by sequencing pooled samples. BMC Bioinformatics.

[21] Bickel PJ, Ritov Y, Rydén T (1998). Asymptotic normality of the maximum-likelihood estimator for general hidden Markov models. Ann Stat.

[22] Chen H, Xing H, Zhang N (2011). Estimation of parent specific DNA copy number in tumors using high-density genotyping arrays. PLoS Comput Biol.

[23] Wang K, Li M, Hadley D, Liu R, Glessner J, Grant S (2007). PennCNV: An integrated hidden Markov model designed for high-resolution copy number variation detection in whole-genome SNP genotyping data. Genome Res.

[24] Browning SR, Browning BL (2007). Rapid and accurate haplotype phasing and missing data inference for whole genome association studies by use of localized haplotype clustering. Am J Hum Genet.

[25] Baum L, Petrie T, Soules G, Weiss N (1970). A maximization technique occurring in the statistical analysis of probabilistic functions of Markov chains. Ann Math Stat.

[26] Dempster A, Laird N, Rubin D (1977). Maximum likelihood from incomplete data via the EM algorithm (With discussion). J R Stat Soc Ser B.

[27] Rabiner L (1989). A tutorial on Hidden Markov models and selected applications in speech recognition. Proc IEEE.

[28] Gualdrón Duarte J, Bates R, Ernst C, Raney N, Cantet R, Steibel J (2013). Genotype imputation accuracy in a F2 pig population using high density and low density SNP panels. BMC Genet.

[29] Choi I, Bates R, Raney N, Steibel J, Ernst C (2012). Evaluation of QTL for carcass merit and meat quality traits in a US commercial Duroc population. Meat Sci.

[30] Choi I, Steibel J, Bates R, Raney N, Rumph J, Ernst C (2011). Identification of Carcass and Meat Quality QTL in an F(2) Duroc x Pietrain pig resource population using different least-squares analysis models. Front Genet.

[31] Edwards D, Ernst C, Raney N, Doumit M, Hoge M, Bates R (2008). Quantitative trait loci mapping in an F2 Duroc x Pietrain resource population. I. Growth traits. J Anim Sci..

[32] Edwards D, Ernst C, Raney N, Doumit M, Hoge M, Bates R (2008). Quantitative trait locus mapping in an F2 Duroc x Pietrain resource population. II. Carcass and meat quality traits, J Anim Sci.

[33] Badke Y, Bates R, Ernst C, Schwab C, Steibel J (2012). Estimation of linkage disequilibrium in four US pig breeds. BMC Genomics.

[34] Steibel J, Bates R, Rosa G, Tempelman R, Rilington V, Ragavendran A (2011). Genome-wide linkage analysis of global gene expression in loin muscle tissue identifies candiyear genes in pigs. PLoS One.

[35] Ramos A, Crooijmans R, Affara N, Amaral A, Archibald A, Beeyer J (2009). Design of a high density SNP genotyping assay in the pig using SNPs identified and characterized by next generation sequencing technology. PLoS One.

[36] Trapnell C, Pachter L, Salzberg SL (2009). TopHat: discovering splice junctions with RNA-Seq. Bioinformatics.

[37] Langmead B, Salzberg SL (2012). Fast gapped-read alignment with Bowtie 2. Nat Methods.

[38] Li H, Handsaker B, Fennell T, Ruan J, Homer N, Marth G (2009). The sequence alignment/Map format and SAMtools. Bioinformatics.

[39] Griffiths DA (1973). Maximum likelihood estimation for the beta-binomial distribution and an application to the household distribution of the total number of cases of disease. Biometrics.

[40] McLaren W (2010). Deriving the consequences of genomic variants with the Ensembl API and SNP effect predictor. Bioinformatics.

