# Supplementary material for: A hidden Markov approach for ascertaining cSNP genotypes from RNA sequence data in the presence of allelic imbalance by exploiting linkage disequilibrium
Source: BMC Bioinformatics. 2015 Feb 22;16:61. doi: 10.1186/s12859-015-0479-2 (PMC4351697; doi:10.1186/s12859-015-0479-2)
Supplement: Additional file 1 — Supplemental. Contains a detailed algorithm of the proposed HMM-ASE algorithm (Section 1), with an extension to the case with distance dependent transition matrix (Section 2). In Section 3, we include some additional real data analysis results using distance dependent transition matrix and the comparison with BCFtools and Beagle after excluding the zero counts in the data set. In Section 4, two additional tables for simulation studies with haplotypes generated from the real data are also included. [file 12859_2015_479_MOESM1_ESM.pdf]

Supplemental Material: A Hidden Markov Approach for Ascertaining SNP Genotypes from Next Generation Sequencing Data in the Presence of Allelic Imbalance by Exploiting Linkage Disequilibrium

Juan P. Steibel, Heng Wang and Ping-Shou Zhong*

Department of Animal Science, Lyman Briggs College and Department of Statistics and Probability, Michigan State University

1. Details of the EM Algorithm and its Implementation

In this section, we provide details of the EM algorithm for obtaining the maximum likelihood estimates (MLE) of $\boldsymbol{\theta}$ where $\boldsymbol{\theta} = (\alpha_1, \beta_1, \alpha_2, \beta_2, e, \mathbf{A})^T$, where $\mathbf{A} = (a_{kk'})_{k,k'=1,\cdots,M}$ are parameters in the transition matrix.

To this end, we introduce the following complete data corresponding the observed data $\mathbf{X}$,

$$\mathbf{Y} = \{G_{il}, \delta_{il}, \mathbf{X}_{il} : l = 1, \cdots, L\} \text{ for } i = 1, \cdots, n.$$

The likelihood function for the complete data is

$$L(\boldsymbol{\theta}|\mathbf{Y}) = f(\mathbf{Y}|\boldsymbol{\theta}) = f(\mathbf{X}|\mathbf{G})f(\mathbf{G}|\boldsymbol{\theta}) = \prod_{i=1}^{n}\prod_{l=1}^{L} f_X(\mathbf{X}_{il}|G_{il}) \prod_{i=1}^{n}\prod_{l=2}^{L} a_{g_{i(l-1)},g_{il}}(\boldsymbol{\theta})\pi_{g_{i1}}(\boldsymbol{\theta}).$$

*The authors acknowledge the support from NSF grants DMS-1209112, DMS-1309156, NIFA-AFRI 2010-65205-20342 and NIFA-AFRI 2011-07015-30338.

where $f_X(\mathbf{X}_{il}|G_{il},\delta_{il})$ is the conditional density of $\mathbf{X}_{il}$. It follows that the log-likelihood function of $L(\boldsymbol{\theta}|\mathbf{Y})$ is given by

$$\log L(\boldsymbol{\theta}|\mathbf{Y}) = \sum_{i=1}^{n}\sum_{l=1}^{L}\log f_X(\mathbf{X}_{il}|G_{il}) + \sum_{i=1}^{n}\sum_{l=2}^{L}\log\{a_{g_{i(l-1)},g_{il}}(\boldsymbol{\theta})\} + \sum_{i=1}^{n}\log\{\pi_{g_{i1}}(\boldsymbol{\theta})\}.$$

Define $\mathcal{L}_{i,k}(l)$ as

$$\begin{aligned}\mathcal{L}_{i,k}(l) := P(G_{il}=k|\mathbf{X}) &= \sum_{G_i} P(G|\mathbf{X})I(G_{il}=k) \\ &= \sum_{G_i}\frac{P(\mathbf{X},G)}{P(\mathbf{X})}I(G_{il}=k). \qquad (1.1)\end{aligned}$$

and

$$H_{i,k,k'}(l) = P(G_{il}=k, G_{i(l+1)}=k'|\mathbf{X}) = \sum_{G_i} P(G_i|\mathbf{X})I(G_{il}=k)I(G_{i(l+1)}=k').$$

The conditional expection of $\log L(\boldsymbol{\theta}|\mathbf{Y})$ given $\mathbf{X}$ evaluated at $\boldsymbol{\theta}^{(m-1)}$ is

$$\begin{aligned}E\{\log L(\boldsymbol{\theta}|\mathbf{Y})|\mathbf{X},\boldsymbol{\theta}^{(m-1)}\} &= \sum_{i=1}^{n}\sum_{k=1}^{M}\mathcal{L}_{i,k}(1)\log(\pi_k(\boldsymbol{\theta})) \\ &+ \sum_{i=1}^{n}\sum_{l=2}^{L}\sum_{k=1}^{M}\sum_{k'=1}^{M} H_{i,k,k'}(l)\log(a_{k,k'}(\boldsymbol{\theta})) \\ &+ \sum_{i=1}^{n}\sum_{l=1}^{L}\sum_{k=1}^{M}\mathcal{L}_{i,k}(l)\log f_X(\mathbf{X}_{il}|G_{il}=k,\boldsymbol{\theta})\end{aligned}$$

where we used $E_{\delta_{il}|G_{il}=k}\{\log f_X(\delta_{il}|G_{il}=k,\boldsymbol{\theta})\}=0$.

We then maximize $E\{\log L(\boldsymbol{\theta}|\mathbf{Y})|\mathbf{X},\boldsymbol{\theta}^{(m-1)}\}$ with respect to $\boldsymbol{\theta}$, say, the maximal is taken at point $\boldsymbol{\theta}^{(m)}$. We updated the parameter $\boldsymbol{\theta}^{(m-1)}$ by $\boldsymbol{\theta}^{(m)}$. It can be shown, by a constrained maximization, that $a_{kk'}^{(m)}$ are

$$a_{kk'}^{(m)} = \frac{\sum_{i=1}^{n}\sum_{l=1}^{L} H_{i,k,k'}(l)}{\sum_{i=1}^{n}\sum_{l=1}^{L}\sum_{k'=1}^{M} H_{i,k,k'}(l)} = \frac{\sum_{i=1}^{n}\sum_{l=1}^{L} H_{i,k,k'}(l)}{\sum_{i=1}^{n}\sum_{l=1}^{L}\mathcal{L}_{i,k}(l)} \qquad (1.2)$$

and $\alpha_1^{(m)}, \beta_1^{(m)}, \alpha_2^{(m)}, \beta_2^{(m)}, e^{(m)}$ satisfying

$$\sum_{i=1}^{n}\sum_{l=1}^{L}\sum_{k=1}^{M}\mathcal{L}_{i,k}(l)\frac{\partial \log f_X(\mathbf{X}_{il}|G_{il}=k;\boldsymbol{\theta})}{\partial(\alpha_1,\beta_1,\alpha_2,\beta_2,e)}=0 \tag{1.3}$$

where the marginal probability mass function of $\mathbf{X}_{il}$ given G_{il} is

$$f_X(\mathbf{X}_{il}|G_{il}=g;\boldsymbol{\theta})=\begin{cases}\binom{n_{il}}{\mathbf{X}_{il}}(1-e)^{X_{il1}}(\frac{e}{3})^{X_{il2}+X_{il3}+X_{il4}} & \text{for } g=1\\ \binom{n_l}{\mathbf{X}_{il}}(0.5-\frac{e}{3})^{X_{il1}+X_{il4}}(\frac{e}{3})^{X_{il2}+X_{il3}} & \text{for } g=2\\ \binom{n_l}{\mathbf{X}_{il}}(\frac{e}{3})^{X_{il2}+X_{il3}}\frac{C_0(\boldsymbol{\theta};X_{il1},X_{il4})}{0.5^{\alpha_1+\beta_1-1}\mathbf{B}(\alpha_1,\beta_1)} & \text{for } g=3\\ \binom{n_l}{\mathbf{X}_{il}}(\frac{e}{3})^{X_{il2}+X_{il3}}\frac{C_1(\boldsymbol{\theta};X_{il1},X_{il4})}{0.5^{\alpha_2+\beta_2-1}\mathbf{B}(\alpha_2,\beta_2)} & \text{for } g=4\\ \binom{n_t}{\mathbf{X}_{il}}(1-e)^{X_{il4}}(\frac{e}{3})^{X_{il1}+X_{il2}+X_{il3}} & \text{for } g=5\end{cases}$$

where $\binom{n_{il}}{\mathbf{X}_{il}}=\frac{n_l!}{X_{il1}!X_{il2}!X_{il3}!X_{il4}!}$,

$$C_0(\boldsymbol{\theta};X_{il1},X_{il4})=\int_{0.5}^{1}((1-\frac{4e}{3})\delta+\frac{e}{3})^{X_{il1}}((\frac{4e}{3}-1)\delta+1-e)^{X_{il4}}(1-\delta)^{\alpha_1-1}(\delta-0.5)^{\beta_1-1}d\delta$$

and

$$C_1(\boldsymbol{\theta};X_{il1},X_{il4})=\int_{0}^{0.5}((1-\frac{4e}{3})\delta+\frac{e}{3})^{X_{il1}}((\frac{4e}{3}-1)\delta+1-e)^{X_{il4}}\delta^{\alpha_1-1}(0.5-\delta)^{\beta_1-1}d\delta.$$

The details of the implementation of above EM algorithm can be done by a forward and backward method. The following forward-backward algorithm implements the EM algorithm in three steps:

1. Compute $\alpha_{i,k}$ (forward probabilities), $\beta_{i,k}$ (backward probabilities) and $P_{\mathbf{X}_i}$.

$$\alpha_{i,k}(1)=\pi_k f_X(\mathbf{X}_{i1}|G_{il}=k;\alpha,\beta,e) \quad \text{for all } 1\le k\le M \text{ and } 1\le i\le n;$$

$$\alpha_{i,k}(l)=f_X(\mathbf{X}_{il}|G_{il}=k;\alpha,\beta,e)\sum_{k'=1}^{M}\alpha_{i,k'}(l-1)a_{k',g} \quad \text{for } 1<l\le L \text{ and } 1\le i\le n;$$

$$\beta_{i,k}(M)=1 \quad \text{for all } 1\le k\le M \text{ and } 1\le i\le n;$$

$$\beta_{i,k}(l)=\sum_{k'=1}^{M}a_{g,k'}f_X(\mathbf{X}_{i(l+1)}|G_{i,(l+1)}=k';\alpha,\beta,e)\beta_{i,k'}(l+1)$$

$$\text{for } 1\le l<L,\ 1\le k\le M \text{ and } 1\le i\le n;$$

and $P_{\mathbf{X}_i} = \sum_{k=1}^{M} \alpha_{i,k}(1)\beta_{i,k}(1)$.

2. Compute $\mathcal{L}_{i,k}(l)$ and $H_{i,k,k'}(l)$ using $\alpha_{i,k}(l)$ and $\beta_{i,k}(l)$.

$$\mathcal{L}_{i,k}(l) = \frac{\alpha_{i,k}(l)\beta_{i,k}(l)}{P_{\mathbf{X}_i}}$$
$$H_{i,k,k'}(l) = \alpha_{i,k}(l)a_{kk'}f_X(\mathbf{X}_{i(l+1)}|G_{i(l+1)} = k'; \alpha, \beta, e)\beta_{i,k'}(l+1).$$

3. Update parameters $\boldsymbol{\theta} = (a_{k,l}, \alpha_1, \beta_1, \alpha_2, \beta_2, e)^T$ by

$$\sum_{i=1}^{n}\sum_{l=1}^{L}\sum_{k=1}^{M} \mathcal{L}_{i,k}(l)\frac{\partial \log f_X(\mathbf{X}_{il}|G_{il} = k; \alpha, \beta, e)}{\partial \boldsymbol{\theta}} = 0. \tag{1.4}$$

4. Repeat Step 1-3 until all the parameters $\boldsymbol{\theta}$ converge.

Since there is no closed form integration $C_0(\boldsymbol{\theta}; X_{il1}, X_{il2})$ and $C_1(\boldsymbol{\theta}; X_{il1}, X_{il2})$, we compute them using a numerical integration. To update parameters α, β, e in (1.4), we define $\hat{e}^{(m)}$ as the solution of

$$\begin{aligned}
0 \;=\; & \sum_{i=1}^{n}\sum_{l=1}^{L}\Big\{\mathcal{L}_{i,1}(l)(-\frac{X_{il1}}{1-e} + \frac{n_{il} - X_{il1}}{e}) + \mathcal{L}_{i,2}(l)(-\frac{X_{il1} + X_{il4}}{1.5 - e} + \frac{X_{il2} + X_{il3}}{e}) \\
& + \mathcal{L}_{i,3}(l)\Big\{\frac{X_{il2} + X_{il3}}{e} + \frac{\partial C_0(\hat{\alpha}_1^{(m-1)}, \hat{\beta}_1^{(m-1)}, e; X_{il1}, X_{il4})/\partial e}{C_0(\hat{\alpha}_1^{(m-1)}, \hat{\beta}_1^{(m-1)}, e; X_{il1}, X_{il4})}\Big\} \\
& + \mathcal{L}_{i,4}(l)\Big\{\frac{X_{il2} + X_{il3}}{e} + \frac{\partial C_1(\hat{\alpha}_2^{(m-1)}, \hat{\beta}_2^{(m-1)}, e; X_{il1}, X_{il4})/\partial e}{C_1(\hat{\alpha}_2^{(m-1)}, \hat{\beta}_2^{(m-1)}, e; X_{il1}, X_{il4})}\Big\} \\
& + \mathcal{L}_{i,5}(l)(-\frac{X_{il4}}{1-e} + \frac{n_{il} - X_{il4}}{e})\Big\}
\end{aligned}$$

and $\hat{\alpha}_s^{(m)}, \hat{\beta}_s^{(m)}$ $(s = 1, 2)$ as the solutions to the following two equations:

$$\begin{aligned}
0 \;=\; & \sum_{i=1}^{n}\sum_{l=1}^{L} \mathcal{L}_{i,2+s}(l)(-\log 2 - \mathbf{B}^{-1}(\alpha_s, \hat{\beta}_s^{(m-1)})\frac{\partial \mathbf{B}(\alpha_s, \hat{\beta}_s^{(m-1)})}{\partial \alpha_s} \\
& + C_{s-1}^{-1}(\alpha_s, \hat{\beta}_s^{(m-1)}, \hat{e}^{(m-1)}; X_{il1}, X_{il4})\frac{\partial C_{s-1}(\alpha_s, \hat{\beta}_s^{(m-1)}, \hat{e}^{(k-1)}; X_{il1}, X_{il4})}{\partial \alpha_s})
\end{aligned}$$

$$
\begin{aligned}
0 &= \sum_{i=1}^{n}\sum_{l=1}^{L}\mathcal{L}_{i,2+s}(l)(-\log 2-\mathbf{B}^{-1}(\hat{\alpha}_s^{(m-1)},\beta_s)\frac{\partial\mathbf{B}(\hat{\alpha}_s^{(m-1)},\beta_s)}{\partial\beta_s}\\
&\quad +C_{s-1}^{-1}(\hat{\alpha}_s^{(m-1)},\beta_s,\hat{e}^{(m-1)};X_{il1},X_{il4})\frac{\partial C_{s-1}(\hat{\alpha}_s^{(m-1)},\beta_s,\hat{e}^{(m-1)};X_{il1},X_{il4})}{\partial\beta_s}).
\end{aligned}
$$

2. Transition Probabilities Depending on Distances Among SNPs

In this section, we discuss a generalized version of HMM-ASE, with transition probability taking into consideration of distances among SNPs. The idea is, if the two SNPs are close to each other, it is less likely that the genotype state changes from one SNP to another. While if the two SNPs are far apart, it is more likely that there exists a change on genotype status between the two SNPs. Similar idea was been applied in copy number variation detection by Wang et al. (2013).

If distances among SNPs affect the transition probability, then the transition matrix $\mathbf{A}_l = (a_{kk'}(l))$ depending on the location of a SNP, which is a function of the SNP location l, where

$$
\begin{aligned}
a_{kk'}(l) &= P(G_{i,l+1}=k'|G_{i,l}=k)\\
&= \begin{cases} a^*_{kk'}(1-e^{-\rho d_l}) & k\neq k' \\ 1-(\sum_{k\neq k'}a^*_{kk'})(1-e^{-\rho d_l}) & k=k' \end{cases}, \qquad (1.5)
\end{aligned}
$$

for $k, k' = 1, \ldots, 5$ and $l = 2, \ldots, L$. Here d_l represents the genomic distance between the locations of SNP l and SNP $l+1$. The parameter ρ determines the effect of the distance on the transition probabilities ($\rho > 0$). The parameter $a^*_{kk'}$ affects the transition probabilities from state k to state k', besides the effect of distances. Also, there is a constraint that $a^*_{kk'} \in (0, 1)$ and $\sum_{l\neq k} a^*_{kk'} < 1$ for each k. The expectation

of the log-likelihood function is now changed to

$$
\begin{aligned}
&E\{\log L(\boldsymbol{\theta}|\mathbf{Y})|\mathbf{X},\boldsymbol{\theta}^{(k-1)}\}\\
= &\sum_{i=1}^{n}\sum_{k=1}^{M}\mathcal{L}_{i,k}(1)\log(\pi_k)+\sum_{i=1}^{n}\sum_{l=2}^{L}\sum_{k=1}^{M}H_{i,k,k}(l)\log\left(1-\Big(\sum_{k'\neq k}a^*_{kk'}\Big)\Big(1-e^{-\rho d_l}\Big)\right)\\
&+\sum_{i=1}^{n}\sum_{l=2}^{L}\sum_{k\neq k'}^{M}H_{i,k,k'}(l)\log\left(a^*_{kk}\Big(1-e^{-\rho d_l}\Big)\right)\\
&+\sum_{i=1}^{n}\sum_{l=1}^{L}\sum_{k=1}^{M}\mathcal{L}_{i,k}(l)\log f_X(\mathbf{X}_{it}|G_{it}=k,\boldsymbol{\theta})\\
:= &R_1(\pi_k)+R_2(\boldsymbol{a},\rho)+R_3(\boldsymbol{a},\rho)+R_4(\boldsymbol{\theta})
\end{aligned}
$$

where $\boldsymbol{a}^*=(a^*_{12},\ldots,a^*_{M,M-1})$.

We modify the forward-backward algorithm in the last section to accommodate the new model (1.5) on the transition matrix. The changes are summarized in the following (1) change the transition probabilities $a_{k',g}$ in step 1 into $a_{k',g}(l)$ and $a_{k,k'}$ in step 2 into $a_{k,k'}(l)$; (2) in additional to the update for parameters $\alpha_1,\beta_1,\alpha_2,\beta_2,e$ in step 3, we also need to update the parameters $a^*_{k,k'},\rho$, which can be done by using the following method. Equating to zero the derivative of $E\{\log L(\boldsymbol{\theta}|\mathbf{Y})|\mathbf{X},\boldsymbol{\theta}^{(k-1)}\}$ with respect to $a^*_{kk'}$ yields

$$
\begin{aligned}
&\frac{\partial R_2(\boldsymbol{a}^*,\rho)}{\partial a^*_{kk'}}+\frac{\partial R_3(\boldsymbol{a}^*,\rho)}{\partial a^*_{kk'}}\triangleq 0 \quad (k,k'=1,\ldots,M;k\neq k')\\
\Rightarrow\quad &\sum_{i=1}^{n}\sum_{l=2}^{L}\frac{(1-e^{-\rho d_l})H_{i,k,k}(l)}{1-(1-e^{-\rho d_l})\sum_{l\neq k}a^*_{kl}}=\sum_{i=1}^{n}\sum_{l=2}^{L}\frac{H_{i,k,k'}(w)}{a^*_{kk'}} \quad (k,k'=1,\ldots,M;k\neq k')\\
\Rightarrow\quad &\sum_{i=1}^{n}\sum_{l=2}^{L}\frac{H_{i,k,1}(l)}{a^*_{k1}}=\ldots=\sum_{i=1}^{n}\sum_{l=2}^{L}\frac{H_{i,k,M}(l)}{a^*_{kM}}=\sum_{i=1}^{n}\sum_{l=2}^{L}\frac{(1-e^{-\rho d_k})H_{i,k,k}(l)}{1-(1-e^{-\rho d_l})\sum_{k'\neq k}a^*_{kk'}}
\end{aligned}
$$

for $k,k'=1,\ldots,M$ and $k'\neq k$.

For each k ($k=1,\ldots,M$), let $\sum_{i=1}^{n}\sum_{l=2}^{L}\frac{H_{i,k,k'}(l)}{a^*_{kk'}}=h_k$, $k'=1,\ldots,M$, and we

find the value of h_k that maximizes

$$\sum_{i=1}^{n}\sum_{l=2}^{L}\sum_{k=1}^{M} H_{i,k,k}(l)\log\left(1-\Big(\sum_{k'\neq k}\frac{\sum_{i=1}^{n}\sum_{l=2}^{L}H_{i,k,k'}(l)}{h_k}\Big)\Big(1-e^{-\rho d_l}\Big)\right)$$
$$+\sum_{i=1}^{n}\sum_{l=2}^{L}\sum_{k\neq k'}^{M} H_{i,k,k'}(l)\log\left(\frac{\sum_{i=1}^{n}\sum_{l=2}^{L}H_{i,k,k}(l)}{h_k}\Big(1-e^{-\rho d_l}\Big)\right)$$

for each k with ρ initially fixed at its value from the previous EM iteration ($\rho^{(m)}$). Then a new value of $\boldsymbol{a}^*$ can be obtained by $a_{kk'} = \frac{\sum_{i=1}^{n}\sum_{l=2}^{L}H_{i,k,k'}(l)}{h_k}$, $k, k' = 1\ldots, M$, $k' \neq k$. Now, an updated value of ρ can be obtained by directly maximizing $R_2(\boldsymbol{a}^*, \rho) + R_3(\boldsymbol{a}^*, \rho)$ with respect to ρ, using the new $\boldsymbol{a}^*$ value.

3. Additional Results in Real Data Analysis

In this section, we present some additional results from the real data analysis. The following contigency table Table S.1 reports the performance of the HMM-NASE DD and the HMM-ASE DD method, which are, respectively, HMM-NASE and HMM-ASE methods with transition probability matrix depending on distance among adjacent SNPs.

Comparing the results in the following Table S.1 with the results reported in Table 5 in the paper, we found that HMM-NASE DD method produced exactly the same results as the HMM-NASE method. And the HMM-ASE DD method had a higher empirical false positive rate than HMM-ASE method, which might be due to the over parameterization in the HMM-ASE DD model. This indicates that, there is no advantage of using distance dependent transition matrix for SNPs in a small neighborhood.

We also compared with BCF tools and the BEAGLE methods to the actual genotypes after excluding the zero counts data excluded in the HMM-ASE and HMM-NASE. The results are collected in Table S.2. As we can see that the HMM-ASE

Table S.1: Contingency tables of genotype calling with two methods (columns), HMM-NASE Distance Dependent and HMM-ASE DD, versus actual genotypes (rows). Values in bold represent counts of correct calls. The other values are incorrect calls or Non-called (NC).

	HMM-NASE DD			HMM-ASE DD		
Actual	Genotype,Reads>0			Genotype,Reads>0		
genotype	He	Ho	NC	He	Ho	NC
He	**570**	1	20	**571**	0	20
Ho	2	**921**	46	34	**889**	46

and HMM-NASE did slightly better than the BCF tools and the BEAGLE on the non-zero counts.

Table S.2: Contingency tables of genotype calling with two methods (columns), BCF tools and the BEAGLE method, versus actual genotypes (rows). Values in bold represent counts of correct calls. The other values are incorrect calls or Non-called (NC) or with zero counts.

	BCF Tools			BEAGLE		
Actual	Genotype,Reads>0			Genotype,Reads>0		
genotype	He	Ho	NC	He	Ho	NC
He	**565**	5	21	**569**	2	20
Ho	3	**920**	46	4	**919**	46

4. Additional Results in Simulation Studies

Table S.3: The EFDR (homozygous called heterozygous) and EFNR (heterozygous called homozygous) with 10 SNPs using the haplotype structures generated from real data. Their standard deviations shown in the parenthesis.

λ		Number of individuals		
		6	12	24
		EFDR		
8	ASE	0.0562 (0.0522)	0.0835 (0.0616)	0.0761 (0.0348)
	NASE	0.0420 (0.0388)	0.0359 (0.0470)	0.0257 (0.0171)
16	ASE	0.0059 (0.0128)	0.0259 (0.0197)	0.0271 (0.0141)
	NASE	0.0000 (0.0000)	0.0000 (0.0000)	0.0021 (0.0065)
24	ASE	0.0462 (0.0656)	0.0166 (0.0203)	0.0151 (0.0114)
	NASE	0.0000 (0.0000)	0.0000 (0.0000)	0.0000 (0.0000)
32	ASE	0.0090 (0.0201)	0.0050 (0.0115)	0.0152 (0.0169)
	NASE	0.0000 (0.0000)	0.0000 (0.0000)	0.0000 (0.0000)
56	ASE	0.0032 (0.0102)	0.0000 (0.0000)	0.0031 (0.0040)
	NASE	0.0000 (0.0000)	0.0000 (0.0000)	0.0000 (0.0000)
		EFNR		
8	ASE	0.0699 (0.0665)	0.0910 (0.0683)	0.0603 (0.0307)
	NASE	0.2558 (0.0640)	0.2443 (0.1121)	0.2398 (0.0693)
16	ASE	0.0333 (0.0254)	0.0348 (0.0293)	0.0208 (0.0130)
	NASE	0.3017 (0.1594)	0.2524 (0.0917)	0.2061 (0.0547)
24	ASE	0.0202 (0.0317)	0.0150 (0.0150)	0.0165 (0.0104)
	NASE	0.1238 (0.0832)	0.2253 (0.0832)	0.1803 (0.0504)
32	ASE	0.0079 (0.0129)	0.0105 (0.0101)	0.0116 (0.0125)
	NASE	0.1447 (0.1047)	0.1661 (0.0576)	0.1735 (0.0456)
56	ASE	0.0031 (0.0099)	0.0046 (0.0074)	0.0049 (0.0056)
	NASE	0.1092 (0.0505)	0.1637 (0.0845)	0.1582 (0.0560)

Table S.4: The EFDR (homozygous called heterozygous) and EFNR (heterozygous called homozygous) with 10 SNPs using the haplotype structures generated from real data. The data were generated without ASE, namely, all the heterozygous genes has ASE ratio 0.5. Their standard deviations shown in the parenthesis.

λ		Number of individuals		
		6	12	24
		EFDR		
8	ASE	0.0309 (0.0304)	0.0527 (0.0370)	0.0302 (0.0297)
	NASE	0.0345 (0.0285)	0.0403 (0.0334)	0.0292 (0.0289)
16	ASE	0.0000 (0.0000)	0.0071 (0.0138)	0.0016 (0.0034)
	NASE	0.0000 (0.0000)	0.0022 (0.0069)	0.0009 (0.0027)
24	ASE	0.0056 (0.0176)	0.0018 (0.0056)	0.0000 (0.0000)
	NASE	0.0056 (0.0176)	0.0000 (0.0000)	0.0000 (0.0000)
32	ASE	0.0000 (0.0000)	0.0000 (0.0000)	0.0000 (0.0000)
	NASE	0.0000 (0.0000)	0.0000 (0.0000)	0.0000 (0.0000)
56	ASE	0.0000 (0.0000)	0.0000 (0.0000)	0.0000 (0.0000)
	NASE	0.0000 (0.0000)	0.0000 (0.0000)	0.0000 (0.0000)
		EFNR		
8	ASE	0.0419 (0.0431)	0.0280 (0.0145)	0.0479 (0.0262)
	NASE	0.0517 (0.0464)	0.0303 (0.0169)	0.0455 (0.0214)
16	ASE	0.0000 (0.0000)	0.0000 (0.0000)	0.0033 (0.0043)
	NASE	0.0029 (0.0090)	0.0018 (0.0056)	0.0025 (0.0041)
24	ASE	0.0000 (0.0000)	0.0000 (0.0000)	0.0000 (0.0000)
	NASE	0.0000 (0.0000)	0.0000 (0.0000)	0.0000 (0.0000)
32	ASE	0.0000 (0.0000)	0.0000 (0.0000)	0.0000 (0.0000)
	NASE	0.0000 (0.0000)	0.0000 (0.0000)	0.0000 (0.0000)
56	ASE	0.0000 (0.0000)	0.0000 (0.0000)	0.0000 (0.0000)
	NASE	0.0000 (0.0000)	0.0000 (0.0000)	0.0000 (0.0000)

References

Wang, H., Nettleton, D. and Ying, K. (2014) Copy Number Variation Detection Using Next Generation Sequencing Read Counts, BMC Bioinformatics, **15**, 109, doi:10.1186/1471-2105-15-109.
